# Diabetes: A Cinderella Subject We Can’t Afford to Ignore

**DOI:** 10.1371/journal.pmed.1002068

**Published:** 2016-07-05

**Authors:** Juliana C. N. Chan, Andrea O. Y. Luk

**Affiliations:** Department of Medicine and Therapeutics, Hong Kong Institute of Diabetes and Obesity, Li Ka Shing Institute of Health Science, The Chinese University of Hong Kong, Prince of Wales Hospital, Hong Kong Special Administrative Region, China

## Abstract

In a Perspective, Juliana Chan and Andrea Luk discuss the impact of diabetes in countries around the world, setting the scene for a special issue on diabetes prevention comprising discussion pieces and research reports.

## Real-Life Diabetes Stories

Somewhere in Asia, a 60-year-old man is admitted to an overcrowded medical ward with heart failure. He had been diagnosed with type 2 diabetes when presenting with coronary heart disease at the age of 50 and, despite undergoing several coronary interventions, his cardiometabolic risk factors have never been controlled owing to poor drug adherence. In busy outpatient clinics, doctors may have no more than 5 minutes to explain to their patients the nature of diabetes and the importance of self-discipline in diet, behavior and medication adherence, while deciding which drugs to prescribe based on little clinical information. They realize that many patients with hypertension, the metabolic syndrome, or a family history could benefit from screening for diabetes. However, in many settings, doctors can only remind patients to improve their diet and lifestyle and repeat the drug prescriptions. Patients themselves may be unable to afford to wait in long queues in a public clinic or find an insurer to support private outpatient care. Despite possessing some knowledge about diabetes, patients often find it difficult to adopt a suitable level of exercise, take multiple pills, make healthy food choices, and interpret fluctuating blood glucose concentrations, resulting in their diabetes being left unchecked.

## Impact of Diabetes on People and Societies

The many challenges of diabetes prevention and management resonate with patients and their families, and with health care providers, around the world. Of a global population of 7.3 billion, 8.8% of adults (about 415 million) currently have diabetes, and a further 6.7% (318 million) have impaired glucose tolerance (IGT). Of these people, three-quarters are in low-income and middle-income countries [1]. Amongst affected people, over 50% are undiagnosed, with many additional diagnosed patients having their diabetes untreated or uncontrolled [2]. China (109.6 million), India (69.2 million), and the United States (29.3 million) account for 65% of the global population of people with diabetes; despite their small populations, some countries in the Middle East and Pacific Islands have the highest global prevalence, with 20%–40% of people having diabetes [1].

In 2015, globally, about 5 million deaths were due to diabetes, resulting in 1 person dying with diabetes every 6 seconds. Importantly, almost half of the deaths attributable to diabetes occurred in people below the age of 60 years. In many developed and developing countries [1], 1 in 3 people has obesity with or without the metabolic syndrome, substantially increasing their lifetime risk of diabetes, cardiovascular–renal disease, and cancer. On a yearly basis, 3%–10% of people with IGT develop diabetes, and 3%–10% of people with diabetes develop comorbidities including cardiovascular–renal disease, cancer, and dementia [3,4].

## Unique Challenges of Diabetes in the Young and Old

Over 90% of adults with diabetes have type 2 disease, with the highest proportion being middle-aged. Currently, 0.5 million children have type 1 diabetes, with a 3% annual increase in prevalence being especially notable in developing countries, where childhood obesity is unmasking the disease in those genetically predisposed [1,5]. Despite the life-saving nature of insulin in type 1 diabetes, poor access to insulin, accessories (i.e., needles, syringes, and test strips), and therapeutic education continue to cause unnecessary morbidity and premature mortality in developing country populations [6].

In developing countries, 1 in 5 adult persons with type 2 diabetes is diagnosed before the age of 40, and many of these patients have insulin insufficiency due to autoimmune, genetic, and/or epigenetic causes. In addition to childhood obesity, the high prevalence of maternal obesity and gestational diabetes is setting up a vicious cycle of “diabetes begetting diabetes” [7]. About 1 in 7 births is affected by gestational diabetes, this burden mainly falling in low-income and middle-income countries [1]. Compared to their late-onset counterparts, patients with early-onset type 2 diabetes have worse risk factor control and are undertreated with life-saving drugs. Together with poor treatment adherence, often due to lack of knowledge or a disinclination to attend for medical care, many patients reappear with critical illness in middle age, with an average loss of 6 years of life [4,8]. On the other hand, improved survival from cardiovascular disease against a backdrop of chronic hyperglycemia and aging is changing the disease landscape, with frailty, cancer, and dementia emerging as growing and unmet clinical needs exacerbated by comorbid diabetes [9].

## Long-Term Benefits of Prevention and Early Intervention

The enormous burden of diabetes calls for increased efforts in disease prevention, patient empowerment, and risk management. During the last three decades, an enormous body of knowledge has been amassed regarding disease burden, etiologies, pathophysiology, and interventions in type 2 diabetes. Global efforts have discovered genetic causes of diabetes, supporting abnormal beta cell biology as the main culprit, often unmasked by external stressors such as infection, lifestyle, or obesity [10]. In people with IGT, there is evidence that type 2 diabetes can be prevented by modest weight loss and increased physical activity, with the potential to reduce long-term microvascular and macrovascular complications, which can include death. That said, many subjects with genetic predisposition and reduced beta cell reserve may require early drug treatment to control hyperglycemia [11].

In newly diagnosed patients or those with short disease duration, intensified insulin treatment for 2 weeks has been shown to restore insulin secretion with disease remission at 3 years, supporting the concept that early reversal of glucotoxicity could have latent benefits [12]. Similarly, in the United Kingdom Prospective Diabetes Study, a reduction of 0.9% in glycated hemoglobin (A1c) in newly diagnosed patients for 10 years translated to a reduction in all diabetes-related endpoints, including death, 10 years after the trial ended [13]. In high-risk patients with different risk factors and complications, team-based structured care focusing on care processes, attainment of multiple treatment targets, and patient empowerment with feedback can save lives at a manageable cost [14]. The availability of novel blood glucose lowering drugs that do not lead to weight gain and hypoglycemia makes durable glycemic control a real possibility, although economic studies are needed to support such a proposition [15].

## New Hopes and Opportunities in Diabetes Prevention and Care

Despite progress in knowledge about diabetes and advances in therapeutic options, there remain major treatment gaps calling for new perspectives and bespoke solutions for individual populations and settings. A recent meta-analysis indicated that, amongst strategies targeted at patients, care providers, and systems, patient education, task delegation, and team management are most effective in reducing blood glucose, blood lipids, and blood pressure [16]. In high-income countries such as those in Europe and the United States, large scale diabetes prevention programs using team-based approaches with linkage to registries for quality assurance and accountability are now being implemented [17].

In low-income and middle-income countries, insufficient infrastructure, healthcare capacity, and lack of resources make quality improvement programs challenging. Yet by starting with small steps, administrators and doctors can work together to train assistants and redesign workflows to set up registries for risk stratification, care triage, and disease monitoring. In addition to introducing public measures for tobacco control, environmental protection, and regulation of the food and beverage industry, governments must also show leadership to reduce social disparities and ensure access to quality medical care.

Together with academics and other relevant stakeholders, governments should encourage research, train professionals, and create career paths to promote diabetes prevention and holistic care with trust and empathy. By increasing transparency on care protocols, treatment targets and the value of preventive drugs, healthcare providers and payers will be better informed to make rational decisions. To this end, education about biology and common diseases such as diabetes should be a priority to improve health literacy. Through private–public partnerships, innovative care and funding models can be developed to increase the accessibility of health information and essential technology, especially during the early stage of diabetes, when care is least expensive and most affordable. The theme of World Health Day 2016 was “Beat Diabetes” and, after decades of advocacy, commitment, and concerted efforts, there are now promising signs to suggest that diabetes will no longer be the Cinderella of noncommunicable diseases [18].
